# *PSPHL* and breast cancer in African American women: causative gene or population stratification?

**DOI:** 10.1186/1471-2156-15-38

**Published:** 2014-03-20

**Authors:** Seth Rummel, Cayla E Penatzer, Craig D Shriver, Rachel E Ellsworth

**Affiliations:** 1 Clinical Breast Care Project, Windber Research Institute, 620 Seventh Street, Windber, PA 15963, USA; 2 Clinical Breast Care Project, Walter Reed National Military Medical Center, 8901 Wisconsin Avenue, Bethesda, MD 20852, USA; 3 Clinical Breast Care Project, Henry M. Jackson Foundation for the Advancement of Military Medicine, 620 Seventh Street, Windber, PA 15963, USA

**Keywords:** PSPHL, African American, Cancer disparity, Population stratification

## Abstract

**Background:**

Phophoserine phosphatase-like (*PSPHL*) is expressed at significantly higher levels in breast tumors from African American women (AAW) compared to Caucasian women (CW). How overexpression of *PSPHL* contributes to outcome disparities is unclear, thus, molecular mechanisms driving expression differences between populations were evaluated.

**Results:**

PCR was used to detect deletion of 30-Kb of chromosome 7p11 including the first three exons of *PSPHL* using genomic DNA from AAW (199 with invasive breast cancer, 360 controls) and CW (invasive breast cancer =589, 364 controls). Gene expression levels were evaluated by qRT-PCR using RNA isolated from tumor tissue and blood. Data were analyzed using chi-square analysis and Mann–Whitney U-tests; P < 0.05 was used to define significance. Gene expression levels correlated with deletion status: patients homozygous for the deletion had no detectable expression of *PSPHL*, while heterozygous had expression levels 2.1-fold lower than those homozygous for retention of *PSPHL*. Homozygous deletion of *PSPHL* was detected in 61% of CW compared to 6% of AAW with invasive breast cancer (P < 0.0001); genotype frequencies did not differ significantly between AAW with and without breast cancer (P = 0.211).

**Conclusions:**

Thus, deletion of 7p11, which prevents expression of *PSPHL*, is significantly higher in CW compared to AAW, suggesting that this 30-kb deletion and subsequent disruption of *PSPHL* may be a derived trait in Caucasians. The similar frequency of the deletion allele in AAW with and without invasive breast cancer suggests that this difference represent population stratification, and does not contribute to cancer disparities.

## Background

The phosphoserine phosphatase-like (*PSPHL* [GenBank AJ001612.1]) gene was identified over a decade ago as a gene upregulated in fibroblasts from patients with Fanconi Anemia (FA) [1]. Although the gene shares multiple regions of sequence homology with phosphoserine phosphatase, a 476-bp fragment missing from *PSPHL* results in an altered coding region. Microarray analysis of the ocular disease pterygia identified *PSPHL* as one of three genes that could predict disease recurrence; immunohistochemical analysis revealed *PSPHL* is expressed at the superficial epithelium of the primary but not recurrent pterygia, suggesting that decreased *PSPHL* is correlated with increasing alterations in the basement membrane [2]. A deletion/insertion (del/ins) polymorphism within the *PSPHL* gene has also been associated with susceptibility to bipolar disorder [3]. Despite these associations with disease states, the function of *PSPHL* remains unknown.

Microarray analysis has revealed significantly higher *PSPHL* expression levels in a variety of tumor types from African Americans compared to Caucasians. For example, studies using gene expression analysis found significantly higher expression of *PSPHL* in prostate tumors, the surrounding microenvironment, and in primary cell cultures from prostate tumors from African American compared to Caucasian men [4,5]. Higher expression of *PSPHL* has also been found in breast tumors, the tumor microenvironment and non-malignant breast stroma from African American women (AAW) [6,7]. Expression levels of *PSPHL* were also found to be significantly higher in endometrial tumors from AAW compared to Caucasian women (CW) [8,9].

This detection of significantly higher levels of *PSPHL* has been in sex-hormone derived tumors, which have been shown to have less favorable outcomes in African American compared to Caucasian patients [10]. Altered expression of *PSPHL* in FA suggests that *PSPHL* may influence rates of cellular proliferation [1], which may promote more aggressive tumor biology and less favorable outcomes. *PSPHL* is an 841 bp gene spread over four exons on chromosome 7p11.2. The promoter and first three exons of PSPHL are located within a 30 Kb region that is not represented in the human reference sequence, thus individuals harboring this deletion would not express PSPHL. If increased expression of *PSPHL* drives pro-tumorigenic properties, this may contribute to higher mortality rates in AAW. In contrast, baseline expression of *PSPHL* is heritable [11], thus higher expression levels of *PSPHL* may reflect different minor allele frequencies in patients of European compared to African ancestry. To determine whether *PSPHL* is involved in tumorigenesis or reflects population stratification, the association between retention or loss of the 30 Kb region on chromosome 7p11 and expression levels of *PSPHL* were investigated in African American and Caucasian populations.

## Results

Genomic DNA was available from 199 AAW and 589 CW with invasive breast cancer, and 360 AAW and 364 CW controls. Genotypes were successfully generated for all available specimens. All populations were in Hardy-Weinberg equilibrium. Genotype frequencies were significantly different between AAW and CW with breast cancer (Table 1) but not between AAW with and without breast cancer. In addition, genotype frequencies were not significantly different between CW cases and controls. Deletion (del) allele frequencies were significantly lower in AAW with (0.24) and without (0.19) invasive breast cancer compared to those in CW with invasive breast cancer (0.77) and CW controls (0.81).

**Table 1:** Genotype frequencies of the deletion (del)/insertion (ins) polymorphism of 30 Kb on chromosome 7p11 amongst the four patient groups

	**Del/Del**	**Ins/Del**	**Ins/Ins**	**P-value**
AA case	0.06 (n = 12)	0.35 (n = 70)	0.59 (n = 117)	
C case	0.62 (n = 362)	0.34 (n = 202)	0.04 (n = 25)	*P <* 0.0001^a^
AA controls	0.04 (n = 13)	0.31 (n = 112)	0.65 (n = 235)	*P =* 0.211^b^
C controls	0.64 (n = 232)	0.34 (n = 123)	0.02 (n = 9)	*P =* 0.336^c^

AA = African American, C = Caucasian.

^a^P-value calculated between African American and Caucasians with invasive breast cancer.

^b^P-value calculated between African American cases and controls.

^c^P-value calculated between Caucasian cases and controls.

**Figure 1:**
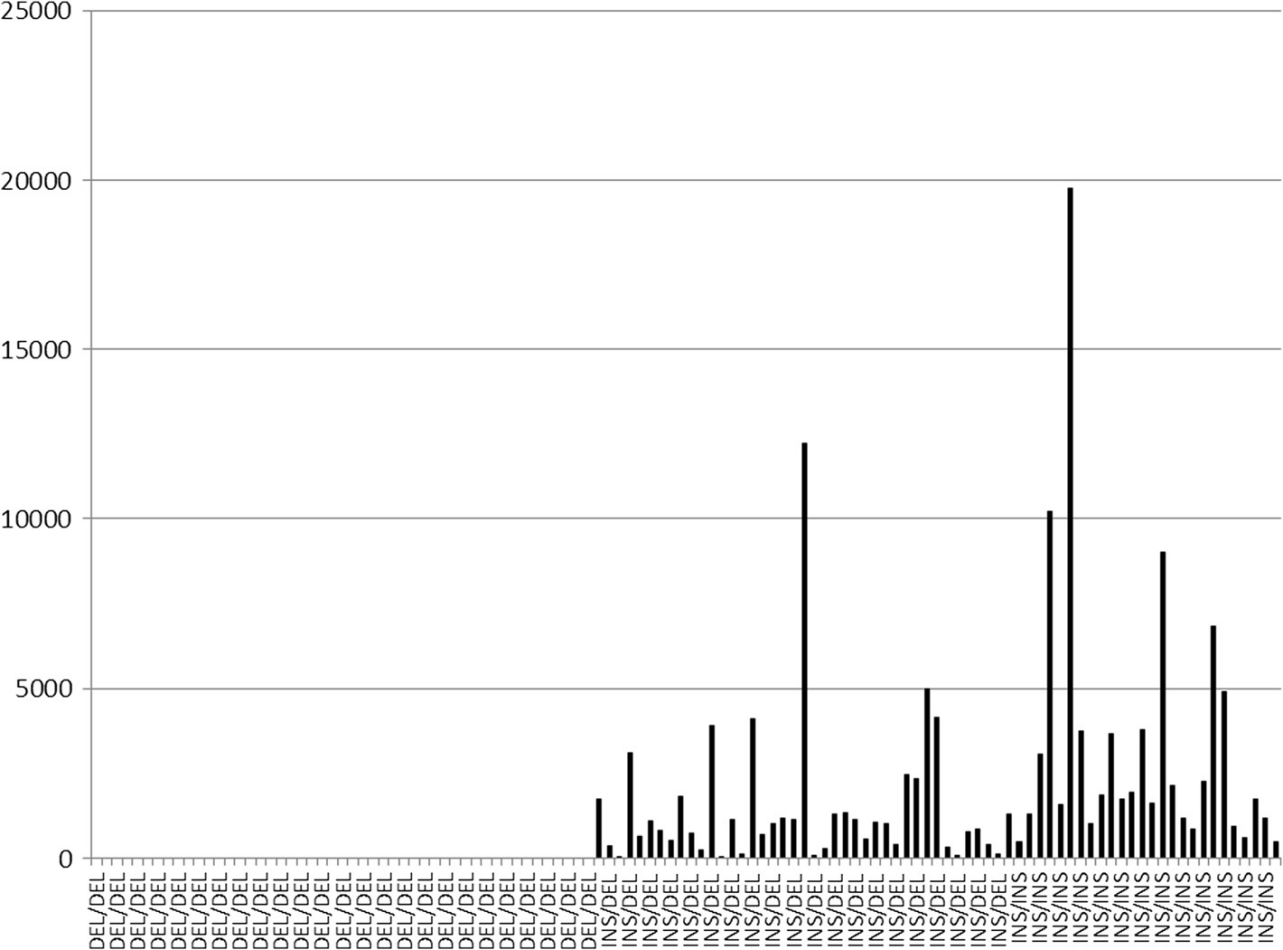
**Graphical representation of expression levels of PHPHL in tumor tissues by genotype.** Del/del = homozygous for deletion variant, del/ins = heterozygous, ins/ins = homozygous for insertion variant.

Expression levels for *PSPHL* were generated by qRT-PCR from 116 tumor specimens (33 AAW and 83 CW) and 49 blood samples, 13 of which overlapped with the tumor samples. Patients with the del/del genotype had no detectable expression in either tissue (n = 49) or blood (n = 23). Expression levels in breast tissue (Figure 1) were 2.1-fold higher in patients homozygous for the insertion allele compared to heterozygotes (*P <* 0.005), while tissue from patients expressing one or two copies of PSPHL were significantly higher than those patients homozygous for the deletion allele (*P <* 0.00001). When analyzing blood RNA, expression of PSPHL was significantly higher in carriers of the insertion allele (ins/ins or ins/del) compared to patients homozygous for the deletion allele (<P < 0.005), although PSPHL levels did not differ significantly in blood RNA between heterozygotes (n = 17) and those with the ins/ins genotype (n = 9), which may be attributable to small sample size.

To determine whether expression of PSPHL was associated with aggressive tumor characteristics or survival, the loss (del/del) or retention (ins/del or ins/ins) of the 30 Kb of chromosome 7p was evaluated within each population for a number of clinical factors (Table 2). Although AAW were more likely to be diagnosed at a younger age, with ER-/HER2-, high-grade tumors than were CW, deletion of PSHPL was not associated with any pathological characteristics.

**Table 2:** Association between retention or absence of 30 Kb region of chromosome 7p and clinicopathological factors by ethnic group

	**AAW**			**CW**		
	**Del/Del (n = 12)**	**Del/Ins or Ins/Ins (n = 186)**	**P-value**	**Del/Del (n = 343)**	**Del/Ins or Ins/Ins (n = 220)**	**P-value**
Age			0.475			0.596
<50 years	0.33	0.44		0.27	0.29	
≥50 years	0.67	0.56		0.73	0.71	
Stage			0.627			0.366
I	0.64	0.55		0.58	0.52	
II	0.36	0.32		0.30	0.32	
III	0.00	0.08		0.10	0.13	
IV	0.00	0.05		0.02	0.03	
ER/HER2 status			0.546			0.458
ER+/HER2-	0.50	0.59		0.71	0.68	
ER+/HER2+	0.17	0.08		0.09	0.12	
ER-/HER2+	0.00	0.06		0.06	0.06	
ER-/HER2-	0.33	0.27		0.14	0.14	
Grade			0.795			0.340
Low	0.18	0.20		0.35	0.28	
Intermediate	0.27	0.36		0.35	0.41	
High	0.55	0.44		0.30	0.31	
Tumor size			0.792			0.367
T1	0.64	0.64		0.73	0.68	
T2	0.36	0.32		0.20	0.26	
T3	0.00	0.04		0.07	0.06	
Lymph node status			0.576			0.550
Positive	0.18	0.26		0.26	0.29	
Negative	0.82	0.74		0.74	0.71	
Status^a^			0.485			0.091
DOC	0.08	0.02		0.02	0.04	
DOD	0.08	0.06		0.07	0.05	
AWD	0.00	0.03		0.04	0.01	
NED	0.84	0.89		0.87	0.9	

^a^DOC = dead other causes, DOD = dead of disease, AWD = alive with disease, NED = no evidence of disease.

## Discussion

AAW have the highest breast cancer mortality rates in the United States with 32.4/100,000 deaths compared to CW who have a mortality rate of 23.9/100,000 [12]. These differences may be attributable to socioeconomic factors such as access to health-care, however, within clinical trials where treatment was standardized, pre- and post-menopausal African American women demonstrated 40% and 50% increased mortality rates, respectively [10]. Microarray analysis has identified a number of genes differentially expressed in tumors from AAW compared to CW, including SOS1, CRYBB2///CRYBB2P1 and PSPHL. The significantly higher expression of *PSPHL* in tumors from African Americans coupled with its putative role in processes such as cellular proliferation may be contributing to more aggressive tumor phenotypes and less favorable outcomes in African Americans. Moreover, if altered expression of *PSPHL* does have a role in tumorigenesis, it becomes a molecular target for the development of new, tailored treatments for the African American population.

Our data demonstrates that the deletion of 30 kb from chromosome 7p11, including the promoter and the first three of four exons of PSPHL, effectively eliminates expression of *PSPHL*. The allele frequency of retention of this region of chromosome 7p is high in AAW, and only 6% of AAW were homozygous for the deletion and thus did not express PSPHL. In contrast, the retention allele frequency was only 21% in CW and 62% of Caucasian cases expressed no *PSPHL*. These data thus provide a functional explanation for expression differences across individuals and between populations. Previous data from our laboratory demonstrated that while within populations, expression levels of *PSPHL* did not differ significantly between tumor and non-malignant stroma, between populations, PSPHL levels were significantly higher in both tumor tissues as well as non-malignant stroma. In addition, higher expression levels of PSPHL in non-malignant tissues from AAW were not associated with higher rates of tumor development [6]. Given the contralateral diagnosis and that none of the other non-malignant patients have progressed to more advanced diagnoses, it is not clear that increased PSPHL is associated with tumorigenesis. In addition, the absence of expression of PSPHL in patients homozygous for the deletion on chromosome 7p was not associated with unfavorable tumor characteristics or survival. In addition to the higher levels of *PSPHL* in a variety of tumor types, the surrounding tumor stroma and non-malignant breast tissues, significantly higher expression of *PSPHL* was also detected in blood endothelial cells from healthy African Americans compared to Caucasians [13]. Together with our data demonstrating that the frequency of the insertion allele did not differ significantly between African American cases and controls, these data suggest that PSPHL expression levels are not associated with breast cancer etiology.

## Conclusions

Differential expression of *PSPHL* provides an example of how population stratification may confound identification of genes associated with health care disparities. Given the higher expression levels of *PSPHL* in breast and prostate tumors from African Americans, a two-gene predictor including *PSPHL* and *CRYBB2* has been proposed to effectively distinguish between tumor epithelia from African American and European Americans [7], however, whether and how these genes play a role in tumor etiology was not determined. Although *PSPHL* is expressed at higher levels in breast (and other) tumors from African Americans, it is also expressed at significantly higher levels in non-tumor tissues as well as blood. In addition, the frequency of the deletion variant which determines expression levels of *PSPHL* is significantly higher in Caucasians but does not differ between African American cases and controls. Thus, differential expression of *PSPHL* caused by retention of a 30 Kb region of 7p11 in individuals of African Ancestry suggests that this difference represent population stratification, and does not contribute to cancer disparities.

## Methods

### Eligibility and enrollment

For inclusion in the Clinical Breast Care Project (CBCP), all patients must have met the following eligibility criteria: 1) adult over the age of 18 years, 2) mentally competent and willing to provide informed consent, and 3) presenting to participating breast centers with evidence of possible breast disease or for routine mammographic screening. Tissue and blood samples were collected with approval from the Walter Reed National Military Medical Center Human Use Committee and Institutional Review Board. All subjects enrolled in the CBCP voluntarily agreed to participate and gave written informed consent. Clinical information was collected for all CBCP samples using questionnaires designed by and administered under the auspices of the CBCP.

### Deletion/insertion polymorphism detection and gene expression analysis

**Figure 2:**
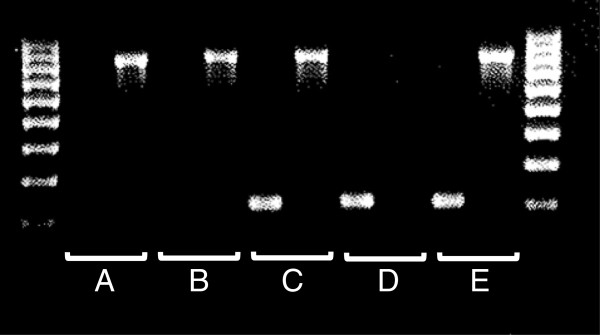
**PCR detection of deletion or insertion of PSPHL gene.** The forward primer that detects the deletion allele spans the deletion breakpoint on chromosome 7p and the primer pair amplifies a 793 bp fragment. The primers that detect the insertion product amplify a 208 bp fragment from exon 1 of PSPHL. Genotypes shown here are **A** = del/del, **B** = del/del, **C** = ins/del, **D** = ins/ins, and **E** = ins/del. Primers used are (all in 5′-3′ direction - insertion forward: AGGCTCCCTGGCTGGC, insertion reverse: CAGGCTCAGGTGAGGCG, deletion forward: AAGCCAGTGCGTCTACAGGTG, deletion reverse: GTGCCAGAAGAACCACACAGTC.

Genomic DNA (AAW invasive = 199, AAW control = 360, CW invasive = 589, CW controls = 364) was isolated from blood clots using the Gentra Clotspin and Puregene DNA purification kits (Qiagen., Valencia, CA). The del/ins polymorphism was assessed using the primer sets described in [3], and the resulting PCR products run on 2% agarose gels (Figure 2). Hardy-Weinberg equilibrium was determined for each population using the OEGE- Online Encyclopedia for Genetic Epidemiology studies Hardy-Weinberg equilibrium calculator (http://www.oege.org/software/hwe-mr-calc.shtml). RNA was isolated from tumor specimens (n = 116) after laser microdissection as previously described [6]. RNA was isolated from blood samples (n = 49) collected in PAXgene Blood RNA tubes (Becton Dickinson, Franklin Lakes, NJ) and isolated using the PAXgene Blood RNA Kit IVD (Qiagen, Valencia, CA) according to manufacturer’s recommendations.

For qRT-PCR, RNA was reverse transcribed using the High-Capacity cDNA Reverse Transcription kit (Life Technologies, Grand Island, NY). qRT-PCR was performed using TaqMan gene expression assay Hs00863464_m1 (Life Technologies, Grand Island, NY). Amplification was performed in duplicate using TaqMan Universal PCR Master Mix (Life Technologies, Grand Island, NY). GAPDH was used as the endogenous control for normalization of all assays. Relative quantification of gene expression levels was determined using the Comparative C_t_ method [14].

### Statistical analysis

Allele and genotype frequencies were compared between AAW with and without invasive breast cancer and between AAW and CW with invasive breast cancer by chi-square analysis using *rxc* Contingency Tables. For qRT-PCR data, the medians of the 2^-ΔΔCt^ values were compared using a Mann–Whitney U test to determine if the relative fold change was significantly different (two-tailed) between genotypes. Significance was determined using *P <* 0.05.

## Competing interest

The authors declare that they have no competing interests.

## Authors’ contributions

SR developed protocols for testing the insertion/deletion, generated gene expression data and participated in the writing of the manuscript, CP genotyped the ins/del variant, CDS oversaw collection of all patient samples and reviewed the manuscript; REE designed the project, performed data analysis and wrote the manuscript. All authors read and approved the final manuscript.

## References

[B1] Planitzer SA Wachl AW Rueckels M Kubbies M Identification of a novel c-DNA overexpressed in Fanconi’s anemia fibroblasts partially homologous to a putative L-3-phosphoserine-phosphatase Gene 1998 210 297 306 10.1016/S0378-1119(98)00083-3 9573387

[B2] Kuo C-H Miyazaki D Nawata N Tominaga T Yamasaki A Sasaki Y Inoue Y Prognosis-determinant candidate genes identified by whole genome scanning in eyes with pterygia Invest Ophthalmol Vis Sci 2007 48 3566 3575 10.1167/iovs.06-1149 17652725

[B3] Akil H Bunney WE Choudary PV Evans EJ Jones EG Li J Lopez JF Lyons DM Molnar M Meyers RM Schatzberg AF Stein R Thompson RC Tomita H Vawter MP Watson SJ Genes and pathways differentially expressed in bipolar disorder and/or major depressive disorder 2005 The Board of Trustees of the Leland Stanford Junior University of Stanford 11158530

[B4] Timofeeva OA Zhang X Ressom HW Varghese RS Kallakruy BV Wang K Ji Y Cheema A Jung M Brown ML Rhim JS Dritschilo A Enhanced expression of SOS1 is detected in prostate cancer epithelial cells from African-American men Int J Oncol 2009 35 751 760 19724911 PMC3727633

[B5] Wallace TA Prueitt RL Yi M Howe TM Gillespie JW Yfantis HG Stephens RM Caporaso NE Loffredo CA Ambs S Tumor immunobiological differences in prostate cancer between African-American and European-American men Cancer Res 2008 68 927 936 10.1158/0008-5472.CAN-07-2608 18245496

[B6] Field LA Love B Deyarmin B Hooke JA Shriver CD Ellsworth RE Identification of differentially expressed genes in breast tumors from African American compared to Caucasian women Cancer 2012 118 1334 1344 10.1002/cncr.26405 21800289

[B7] Martin DN Boersma BJ Yi M Reimers M Howe TM Yfantis HG Tsai YC Williams EH Lee DH Stephens RM Weissman AM Ambs S Differences in the tumor microenvironment between African-American and European-American breast cancer patients Plos ONE 2009 4 e4531 10.1371/journal.pone.0004531 19225562 PMC2638012

[B8] Allard JE Chandramouli GV Stagliano K Hood BL Litzi T Shoji Y Boyd J Berchuck A Conrads TP Maxwell GL Risinger JI Analysis of PSPHL as a candidate gene influencing the racial disparity in endometrial cancer Front Oncol 2012 2 65 22783543 10.3389/fonc.2012.00065 PMC3389395

[B9] Ferguson SE Olshen AB Levine DA Viale A Barakat RR Boyd J Molecular profiling of endometrial cancers from African-American and Caucasian women Gynecol Oncol 2006 101 209 213 10.1016/j.ygyno.2005.11.028 16380157

[B10] Albain KS Unger JM Crowley JJ Coltman CA Hershman DL Racial disparities in cancer survival among randomized clinical trials patients of the southwest oncology group J Natl Cancer Inst 2009 101 984 992 10.1093/jnci/djp175 19584328 PMC2724852

[B11] Morley M Molony CM Weber TM Devlin JL Ewens KG Spielman RS Cheung VG Genetic variation of genome-wide variation in human gene expression Nature 2004 430 743 747 10.1038/nature02797 15269782 PMC2966974

[B12] American Cancer Society Breast cancer facts and figures 2011–2012 2011 Atlanta, GA: American Cancer Society

[B13] Wei P Milbauer LC Enenstein J Nguyen J Pan W Hebbel RP Differential endothelial cell gene expression by African Americans versus Caucasian Americans: a possible contribution to health disparity in vascular disease and cancer BMC Med 2011 9 2 10.1186/1741-7015-9-2 21223544 PMC3029215

[B14] Livak KJ Schmittgen TD Analysis of relative gene expression data using real-time quantitative PCR and the 2(-delta delta C(T)) method Methods 2001 25 402 408 10.1006/meth.2001.1262 11846609

